# ShRNA-Targeted COMMD7 Suppresses Hepatocellular Carcinoma Growth

**DOI:** 10.1371/journal.pone.0045412

**Published:** 2012-09-25

**Authors:** Lu Zheng, Ping Liang, Jing Li, Xiao-bing Huang, Shi-cheng Liu, Hong-zhi Zhao, Ke-qiang Han, Zheng Wang

**Affiliations:** Department of Hepatobiliary Surgery, Xinqiao Hospital, the Third Military Medical University, Chongqing, China; National Cancer Center, Japan

## Abstract

**Background:**

COMMD7 is a newly identified gene overexpressed in hepatocellular carcinoma (HCC) and associated with tumor invasion and poor prognosis. We aim to examine the biological function of COMMD7 in HCC by shRNA silencing.

**Methods:**

COMMD7 expressions were examined in human HCC cell lines HepG2, Huh7, Hep3B, HLE, HLF, SK-Hep-1 and PLC/PRF/5 cells. Recombinant pGenesil-COMMD7-shRNA was transfected into COMMD7-abundant HepG2 cells to silence COMMD7 expression. The effects of COMMD7 silencing on HepG2 cell proliferation in vitro and xenograft tumor growth in vivo were evaluated. Flow cytometry profiling was used to detect the presence of apoptosis in COMMD7-silenced HepG2 cells and to differentiate cell cycle distribution. Electrophoretic mobility shift assay and luciferase reporter assays to examine the activities of nuclear factor-kappaB (NF-κB) signaling pathways in response to tumor necrosis factor (TNF)-α in COMMD7-silenced HepG2 cells.

**Results:**

COMMD7 expression level was abundance in HepG2 and SK-Hep-1 cells. COMMD7 was aberrantly overexpressed in HepG2 cells, whilst pGenesil-COMMD7-shRNA exhibited a maximal inhibition rate of 75%. COMMD7 silencing significantly reduced HepG2 cell proliferation and colony formation. The knockdown of COMMD7 resulted in an increased apoptosis and cell cycle arrest at S-phase. COMMD7 knockdown also exhibited an antineoplastic effect in vivo, which manifested as tumor xenograft growth retardation. COMMD7 silencing also suppressed the responsiveness of NF-κB signaling pathway to the stimulation with TNF-α in vitro. Moreover, the similar suppressive effects of COMMD7 silence on SK-Hep-1 cells were also observed.

**Conclusions:**

COMMD7 contributes to HCC progression by reducing cell apoptosis and overcoming cell cycle arrest. The proliferative and antiapoptotic effects of COMMD7 may be mediated by NF-κB signaling pathway.

## Introduction

Hepatocellular carcinoma (HCC), one of the most common malignancies, prevails worldwide, especially in China. HCC in Chinese population is mainly secondary to viral hepatitis or cirrhosis, and Chinese HCC patients account for approximately half of the global HCC mortalities annually [1]. The incidence of HCC in China still shows an increasing trend due to the pre-existing endemic hepatitis B viral infection although the childhood vaccination plan against HBV has been in place for over two decades. Unfortunately, the clinical outcome and prognosis of HCC are still disappointing because only 10–20% of tumors are resectable at the time of diagnosis, and the five-year survival is generally poor even compared to other gastrointestinal malignancies [2].

As the therapeutic regimen offering the best long-term prognosis, radical hepatectomy that preserves sufficient liver function reserve, especially in cirrhotic patients, remains the first-line treatment of option in current practice. However, curative resection is not applicable in the majority of patients due to extensive intrahepatic disease and/or the compromised liver function [3]. The recurrence rate is expected to be 50–60% following radical resection [4]. Some adjuvant therapies have been available and relatively effective for some treatment-naïve or relapsing patients, including transcatheter arterial chemoembolization [5], radiofrequency ablation [6], selective internal radiation therapy [7], high intensity focused ultrasound [8], and targeted therapy (Sorafenib on trial) [9], usually given in a combined regimen.

Gene therapy has been emerging as a promising intervention against HCC. Multiple oncogenes, suppressor genes, and other regulating genes have been implicated in the pathogenesis of HCC, such as N-ras, c-fos, c-myc, IGF-II, C-erb-2, p53, p16, PTEN, MXR7, KAI1, and HCCA1 [10]. However, due to the complexity of signaling pathways that initiate and maintain the occurrence and progression of HCC through a less understood mechanism, the identification of new target gene that is effective and specific has been always required to advance genetic treatment of HCC.

Using the suppression subtractive hybridization, we identified a novel cDNA fragment (447 bp) highly expressed in human HCC specimens [11]. Further analysis of its sequence and the comparison of its homology shows that one of its poly-A tailed 3′ expressed sequence tags (EST, GenBank ID 694447) is a partial (63%) homolog to a gene sequence at a length of 1476 bp cloned from brain tissues (GenBank ID BC047440) [12]. With the rapid amplification of cDNA 3′-ends (3′RACE), the cloning of the full-length cDNA sequence from the EST of interest confirms its homology to COMMD7 gene located at 20q11.22, which encodes a 200-amino acid cytoplasmic protein [13]. Our clinicopathological analysis has shown that COMMD7 is overexpressed in HCC and associated with advanced tumor staging and portal vein invasion, suggesting a poor prognosis in HCC patients [14]. Moreover, our preliminary study with COMMD7 antisense eukaryotic expression vector showed that COMMD7 silencing inhibited human HepG2 cell growth both in vitro [15]. The anti-proliferative effect of COMMD7 silencing may be attributed to the impairment of cell proliferation and/or survival in an unknown molecular mechanism.

Nuclear factor-kappaB (NF-κB) signaling pathway plays a vital role in bridging chronic active hepatitis with HCC [16]. The transfection of hepatocytes with inhibitory IKKβ suppresses the activation of NF-κB in Mdr2-knockout mice. Furthermore, the inactivation of NF-κB downregulates anti-apoptotic factors, such as A1/Bfl1, c-IAP1 and GADD45β, by activating JNK and c-Jun pathways, and results in the substantive apoptosis of HCC cells [17]. It has been demonstrated the functional domains of COMMD7-encoded amino acids and the involvement of copper metabolism gene MURR1, a regulator of NF-κB activation and translocation [18]. Additionally, c-myc, a common downstream molecule of NF-κB, exhibits a substantially reduced transcription in HepG2 cells subjected to COMMD7 siRNA [19]. These findings suggest that NF-κB signaling may be involved in the biological activity of COMMD7 in HCC.

In this study, we aim to examine the effects of COMMD7 silencing on HCC cell proliferation in vitro and tumor growth in vivo. Additionally, we evaluate the cell apoptosis and cell cycle in COMMD7-knockdown HepG2 cells. Finally, we elaborate whether NF-κB signaling is involved in the biological activities of COMMD7 in the context of HCC.

## Materials and Methods

### Cell lines and Cell Culture

Human HCC cell lines HepG2, Huh7 and Hep3B, as well as human hepatocyte L02, were purchased from American Type Culture Collection (Manassas, VA). Cells were cultured in antibiotics-free Dulbecco’s modified Eagle medium (DMEM)-high glucose (Invitrogen, Carlsbad, CA) containing 10% fetal bovine serum (Invitrogen) at 37°C and at an atmosphere of 5% CO_2_. The culture media were refreshed every three days.

### RT-PCR and Western Blotting Assays of COMMD7

At 80% confluency, cells were dissociated with 0.25% trypsin (Invitrogen) and collected for reverse transcription polymerase chained reaction (RT-PCR) and Western blotting assays with COMMD7. The total RNA was isolated using TRIzol reagent (Invitrogen). Primers of COMMD7 and β-actin (internal control) were synthesized by Invitrogen with the assistance of Primer Premier5.0 (PREMIER Biosoft International, Palo Alto, CA). The sequences were described in Table 1. PCR was performed in a DNA thermal cycler (Applied Biosystems, Carlsbad, CA) in the following condition: one cycle at 94°C for 2 min; 26 cycles, at 94°C for 30 sec, at 62°C for 30 sec, and at 72°C for 45 sec; and one cycle at 72°C for 10 min. PCR products were electrophoresed on 1.5% agarose gel containing 0.5 µg/ml ethidium bromide and visualized using an ultraviolet spectroscopy.

**Table 1:** COMMD7 shRNA sequences and Primer pairs for polymerase chain reaction.

Gene	Primer Sequence (5′ to 3′)		Size of PCR Product (bp)
	Sense	Anti-sense	
COMMD7 shRNA	GATCCGCTCTGGGTCTTAGTGAGGATTCAAGACGTCCTCACTAAGACCCAGAGTTTTTTGTCGACA	GCGAGACCCAGAATCACTCCTAAGTTCTGCAGGAGTGATTCTGGGTCTCAAAAAACAGCTGTTCGA	
COMMD7	AGTGGCTTTCTCCTCACTAAGACC	GGAAAGATTTCTGGCTCAGCTC	181 bp
β-actin	CCAACCGCGAGAAGATGA	ACCAGAGGCGTACAGGGAT AG	98 bp

Total proteins were extracted using radio-immunoprecipitation assay (RIPA) lysis buffer, and electrophoretically transferred onto a nitrocellulose membrane using Hoefer Semiphor semi-dry blotting apparatus (Pharmacia Biotech, Stockholm, Sweden). The blots were blocked with 5% bovine serum albumin overnight, and incubated with 1∶1,000 polyclonal rabbit-anti-COMMD7 (self-prepared) or 1∶2,500 rabbit-anti-β-actin antibodies (UBC, Beijing, China) at 25°C for 60 min, and further incubated with 1∶15,000 biotin-conjugated goat-anti-rabbit IgG (UBC) at 25°C for 30 min. The antibody binding was detected using 1∶15,000 streptavidin-conjugated alkaline phosphatase (UBC), and the color reaction was developed with 5-bromo-4-chloro-3-indolylphosphate and nitroblue tetrazolium (Sigma, St Louis, MO). The band intensity was quantitated using a calibrated imaging densitometer (Bio-Rad Laboratories, Hercules, CA). The expression intensity of COMMD7 was represented as the ratio of COMMD7 to β-actin. The experiments were performed in duplicate and repeated in triplicate independently.

### Construction of pGenesil-COMMD7-shRNA

COMMD7 cDNA was cloned from human HCC specimens in our previous study as approved by the Institutional Review Board at Xinqiao Hospital, the Third Military Medical University. The sequence of short hairpin RNA (shRNA) targeting COMMD7 was designed by using shRNA Target Finder (Ambion, Austin, TX), including *Bam*H I and *Hind* III sites at its terminals, and described in Table 1. Target shRNA was annealed and directly cloned into EGFP reporter plasmid pGenesil-1 (Genesil Biotechnology, Wuhan, China) and subsequently transformed into competent DH5α *E. coli* (gifted by Dr Y Huang, Chongqing Medical University, Chongqing, China). The transformed colonies were selected by kanamycin resistance. Small samples of recombinant plasmids were characterized by using *Sal* I (Genesil Biotechnology) and sequenced by Invitrogen. The recombinant pGenesil-COMMD7-shRNA exhibited the following multiple cloning sites: *Hind* III – shRNA – *BamH* I – U6 promoter – *EcoR* I – *Sal* I–*Xba* I – *Dra* III. The insertion into the target gene fragment was justified by the presence of a 400-bp DNA band upon the digestion with *Sal*I. The sequencing of the recombinant plasmid confirmed its absolute consistency with the target gene insertion sequence at 333–398 bp.

### COMMD7 Silencing with shRNA Transfection

HepG2 cells were plated onto 6-well plates at a density of 1×10^5^ cells per well. At 60–70% confluency, cells were rinsed with phosphate buffered saline and supplemented with serum-free DMEM four hours preceding the transfection. HepG2 cells were transfected with pGenesil-COMMD7-shRNA, scrambled shRNA (Invitrogen), and blank control, respectively. HepG2 cell were transfected at 37°C and at an atmosphere of 5% CO_2_ for 6 h using Lipofectamine™ 2000 (Invitrogen) at the ratio of 1∶2.5 (plasmid/liposome). The culture media containing liposomes were subsequently substituted by DMEM containing 10% FBS for an additional 48-hour culture. Positive clones were selected in the presence of 400 µg/ml G418 (Sigma) and subsequently maintained in 200 µg/ml G418. Transfected HepG2 cells were examined using a fluorescence microscope (Leica Microsystem, Wetzlar, Germany).

### Fluorescent Real-time Quantitative RT-PCR Assay

The down-regulation of COMMD7 in knockdown HepG2 cells was examined using fluorescent real-time quantitative RT-PCR (qRT-PCR) assay. The expressions of COMMD7 and β-actin mRNA were quantitated using the 2*^−ΔΔCt^* method (*ΔΔC_t_* = [*Ct_COMMD7_*– *Ct_β­-actin_*]_experiment_ – [*Ct_COMMD7_*– *Ct_β­-actin_*]_control_), where *Ct* referred to the mean cycle threshold. The experiments were performed in duplicate and repeated in triplicate independently.

### MTT Assay of Cell Viability

Cells were plated onto 96-well plates and cultured in DMEM containing 10% FBS at 37°C and at an atmosphere of 5% CO_2_ for 24 h, 48 h, 72 h and 96 h, respectively. Each well was supplemented with 20 µl of 5 mg/ml MTT, and incubated at 37°C and at an atmosphere of 5% CO_2_ for 4 h. Following the removal of the supernatant, cells were vortexed in 200 µl dimethyl sulfoxide per well for 10 min. The absorbance was measured at 490 nm using an ultraviolet spectrometer (Beckman Coulter, Brea, CA). The experiments were performed in quadruplicate and repeated in triplicate independently. The growth curve was plotted with absorbance at 490 nm against time.

### Colony Formation Assay

Cells were digested in 0.25% trypsin to reconstitute the single-cell suspension at a density of 0.1×10^6^ cells per ml. Cell suspensions were transferred into 6-well plates (300 cells per well) and incubated at 37°C and at an atmosphere of 5% CO_2_ for 10 d. The supernatants were discarded, and cells were rinsed in PBS for twice and fixed in methanolfor 10 min. The cells were appropriately stained with Giemsa’s solution (AppliChem, St Louis, MO) and allowed to air dry at room temperature. The experiments were triplicated and the numbers of colonies containing more than 50 cells were microscopically counted to calculate the colony formation rate as number of colonies/number of cells ×100%.

### Cell Cycle Profiling with Flow Cytometry Assay

Cells were prepared into a single-cell suspension. Cells were fixed in 70% ethanol at 4°C for 24 h, and incubated in 0.5 mg/ml propidium iodine (Calbiochem, San Diego, CA) for 1 h prior to flow cytometry. An Annexin V/FITC kit (Calbiochem) was used to measure the cell apoptosis using a flow cytometry analyzer (BD Biosciences, San Jose, CA).

### Tumorigenicity Assay in Xenograft Tumor Model

The animal care and use was approved by the Animal Research Committee at Xinqiao Hospital. Nude mice (Center of Laboratory Animal Medicine, the Third Military Medical University) of either sex (n = 45), aged 4–5 weeks, were equally and randomly divvied into three groups to receive the subcutaneous injection with HepG2 cells at a dose of 5×10^6^ cells per animal (n = 15 each group), respectively. The dimensions of xenograft nodules were callipered at a weekly interval for four successive weeks. The volume of tumor was calculated using the following equation: V = L×W^2^ (V = volume; L = length; W = width).

### Immunohistochemical Studies

The xenograft tumor were collected and immediately fixed in 10% formalin. Fixed tissues were embedded in paraffin. 4 µm tissue section were immunostained with antibody against Ki67 (abcam, Cambridge, MA). TUNEL staining was performed by a commercial kit (R&D Systems, Minneapolis, MN).

### Electrophoretic Mobility Shift Assay of NF-κB

Electrophoretic mobility shift assay (EMSA) was performed to examine the nuclear translocation of NF-κB. HepG2 cells were plated onto 6-well plates (1×10^6^ cells per well) and pre-treated with 20 ng/ml tumor necrosis factor (TNF)-α for 1 h. TNF-α-pretreated cells were rinsed in PBS and centrifuged at 4°C. The nuclear proteins were extracted using a nuclear protein isolation kit as instructed by the manufacturer (Beyotime). Two microliter of the sample was incubated with 5 µl ^32^P-labeled NF-κB probe (5′-GTAGGGGACTTTCCGAGCTCGATCCTATG-3′; Invitrogen), 5 µl nuclease-free water, and 2 µl EMSA/gel-shift buffer (5x, Beyotime, Haimen, China) at room temperature for 20 min. The proteins binding to the probes were resolved in 1 µl EMSA/gel-shift buffer (10x) and separated on non-denatured SDS-PAGE.

### Luciferase Reporter Assay of NF-κB

Luciferase reporter assay was performed to quantify the activity of NF-κB signaling. HepG2 cells were transfected with firefly luciferase reporter gene (gifted by Dr S Ghosh, Yale University, New Haven, CT) and Renilla RL-TK (Beyotime) by using Lipofectamine™ for 24 h. Transfected cells were pre-treated with 20 ng/ml TNF-α for 1 h and subsequently cleaved in lysis buffer (Beyotime). Cell lysates were vortexed for 15 sec, and centrifuged at 4°C and 12 000 *g* for 2 min. The activities of firefly and Renilla luciferases in the supernatants were measured using an ultraviolet spectrometer (Beckman). The activity of NF-κB was represented as the ratio of firefly luciferase reading to Renilla luciferase reading.

### Statistical Analysis

All the data were expressed as mean ± SD. The intergroup difference was compared by using one-way analysis of variance followed by Duncan’s test. A *P*-value of less than 0.05 was considered statistically significant.

Additional materials and methods are provided in the Materials and Methods S1.

## Results

### COMMD7 is Overexpressed in HCC Cell Lines

**Figure 1:**
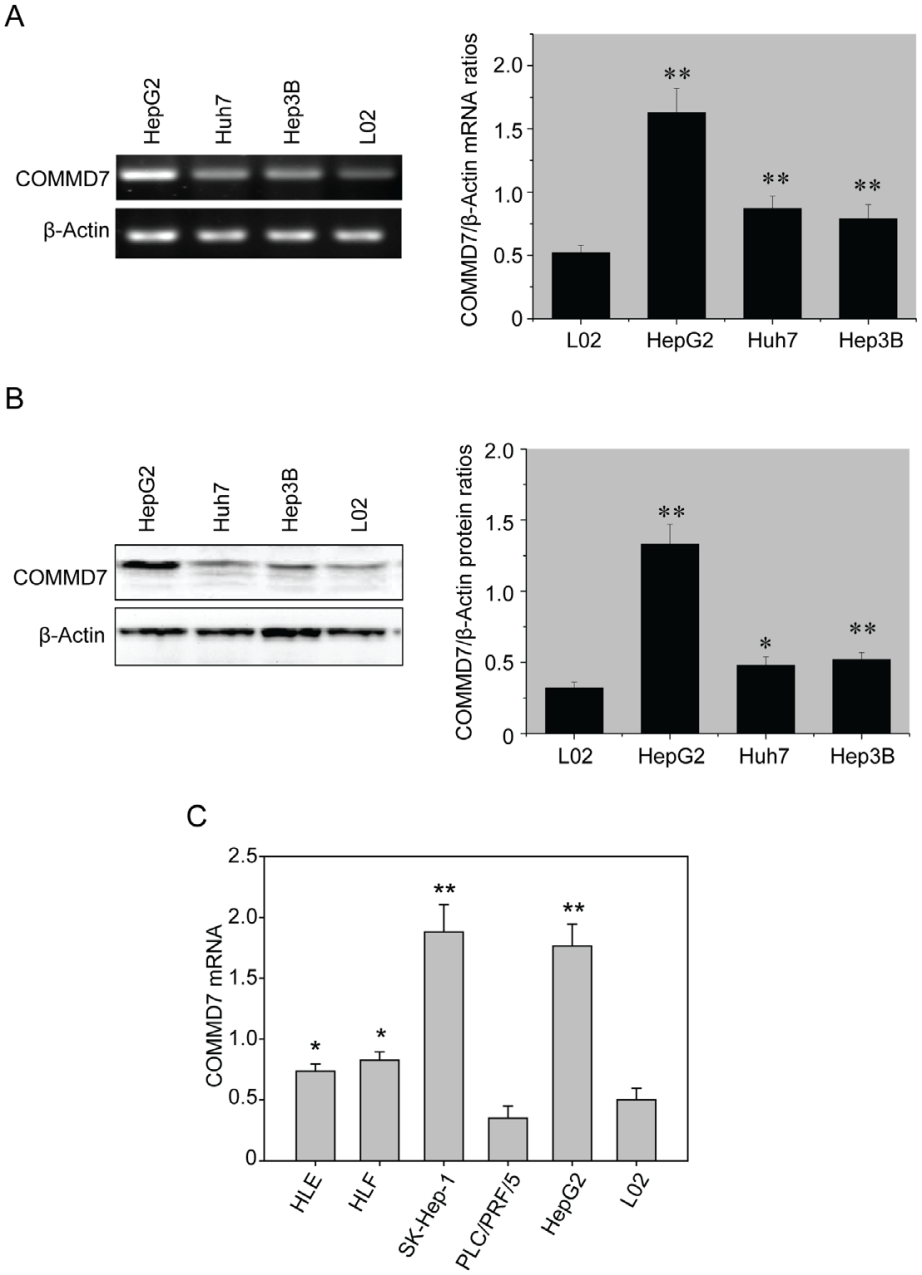
Overexpressions of COMMD7 mRNA and protein in HCC cell lines. (A) RT-PCR assay and (B) Western blotting assay. (C) qRT-PCR of COMMD7 mRNA levels of several HCC cell lines. ^*^ *P*<0.05;^**^ *P*<0.01 vs L02 cells.

On RT-PCR assay, the three HCC cell lines all exhibited an aberrant overexpression of COMMD7 mRNA relative to β-actin as compared to normal hepatocyte cell L02, whereas HepG2 cells expressed a significantly higher level of COMMD7 mRNA than the other two HCC cell lines, namely, Huh7 and Hep3B (HepG2 *vs.* Huh7 *vs.* Hep3B *vs.* L02, 1.613±0.238 *vs* 0.874±0.106 *vs.*0.793±0.116 *vs.* 0.529±0.065, *P*<0.01; Figure 1A). Western blotting analysis further revealed that COMMD7 protein was overexpressed in HepG2 cells as compared to those in Huh7, Hep3B, and L02 cells (1.324±0.140 *vs.* 0.492±0.081 *vs.* 0.519±0.076 *vs.* 0.327±0.034, *P*<0.01; Figure 1B). Given the high abundance of endogenous COMMD7, HepG2 cells were used for the further knockdown experiments. More HCC cell lines, including HLE, HLF, SK-Hep-1 and PLC/PRF/5, were also examined. As shown in Figure 1C, SK-Hep-1 cells expressed an equal level of COMMD7 mRNA as HepG2 cells, and the expression of COMMD7 mRNA in PLC/PRF/5 cells is the same as normal hepatocyte cell L02. Both HLE and HLF showed an increased expression of COMMD7 mRNA.

### COMMD7 shRNA Inhibits HepG2 Cells Proliferation and Colony Formation In Vitro

**Figure 2:**
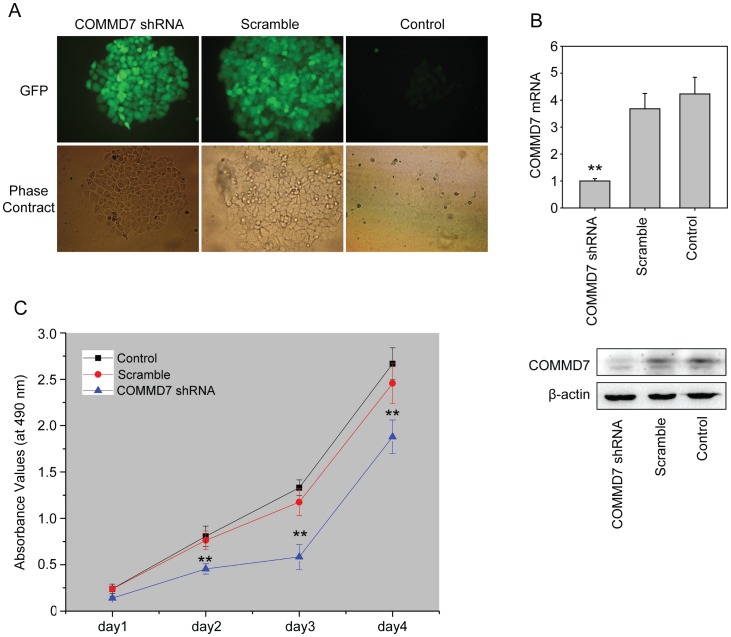
COMMD7 silencing decreased HepG2 cell proliferation. (A) Stably-transfected HepG2 cells on fluorescence microscopy (×200). (B) qRT-PCR quantification and western blotting of COMMD7. (C) MTT assay were performed at indicated days to show the proliferative curve. ^**^ *P*<0.01 vs sramble shRNA treatment.

The selection with G418 detected resistant clones expressed GFP in pGenesil-COMMD7-shRNA and pGenesil-scramble-shRNA treated cells 18 days following G418 maintenance (Figure 2A), whereas control cells became necrotic one week after G418 maintenance. Further qRT-PCR analysis showed that COMMD7 shRNA diminished the endogenous COMMD7 mRNA expression by up to 75%, whereas cells transfected with pGenesil-scramble-shRNA remained comparable to control cells in COMMD7 mRNA expression (Figure 2B). Correspondingly, the protein expression of COMMD7 in COMMD7 shRNA-treated cells was also suppressed.

**Figure 3:**
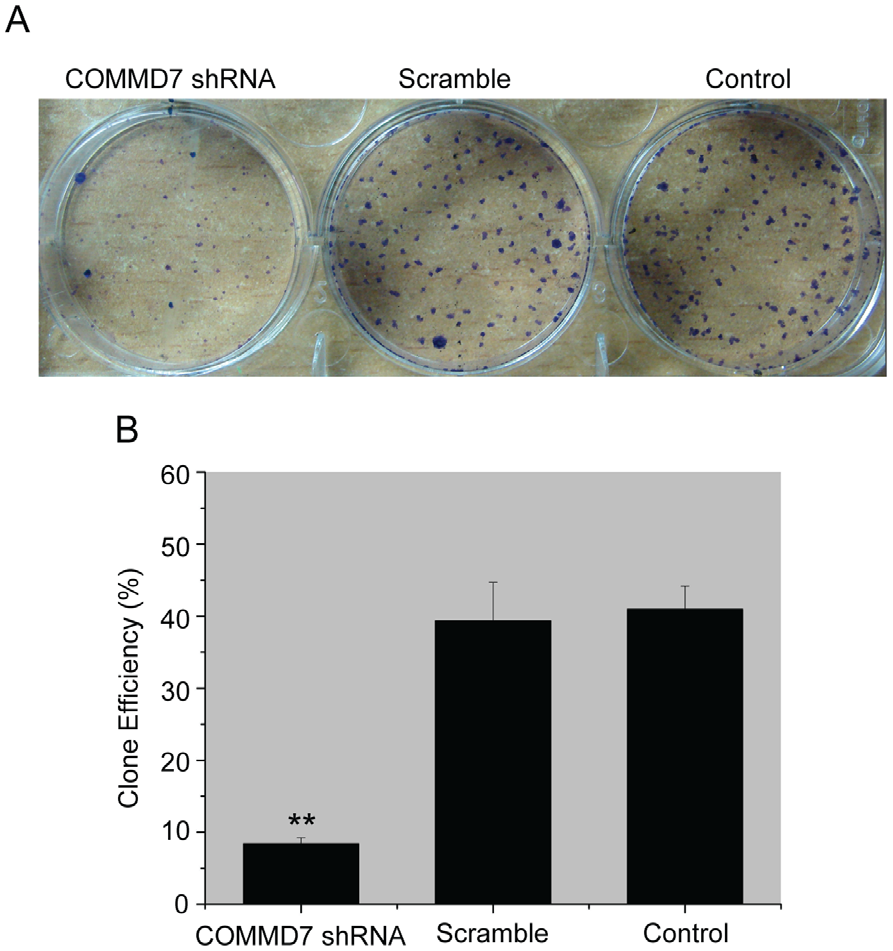
Effects of COMMD7 silencing on HepG2 cell colony formation. (A) Representative photographs of the colony formation assay. (B) Quantification of the colony formation efficiency. ^**^ *P*<0.01 vs sramble shRNA treatment.

COMMD7 shRNA retarded HepG2 cells proliferation in vitro from day 2 as compared to control cells, whereas the proliferation rates remained similar between scramble-shRNA-treated and control cells throughout the culture (Figure 2C). The colony formation experiment also confirmed the reduced proliferative potential in individual COMMD7-silenced HepG2 cells (Figure 3A and B). Since COMMD7 expression was also abundance in SK-Hep-1 cells, we constructed COMMD7 shRNA stably-expressed SK-Hep-1 cells. The suppressive effects of COMMD7 silence on SK-Hep-1 cell proliferation and colony formation were shown in Figure S1A and B.

### COMMD7 shRNA Induces the Apoptosis and Cycle Arrest in HepG2 Cells

**Figure 4:**
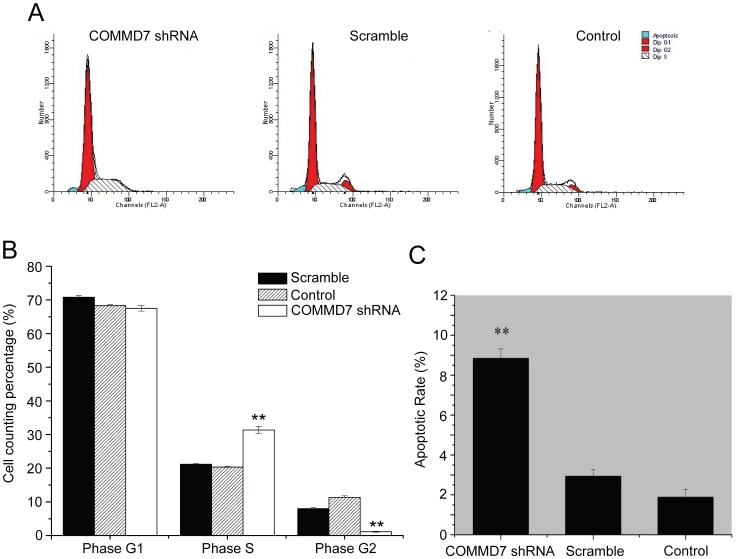
Effects of COMMD7 silencing on HepG2 cell cycle distribution and apoptosis. (A) Representative histograms of flow cytometry. (B) Histogram of percentage of different cell cycle phase. (C) Apoptotic rates were analysed by Annexin V/FITC kit. ^**^ *P*<0.01 vs sramble shRNA treatment.

COMMD7 shRNA significantly increased the fraction of S-phase cells, but decreased that of G2-phase cells in COMMD7 shRNA group as compared to control groups (Figure 4A and B). However, the percentage of G1-phase cells was similar in three groups. Following the COMMD7 silencing, HepG2 cells exhibited a higher apoptotic rate than control cells did (8.9% ±0.5% *vs.* 2.9% ±0.3% *vs.* 1.9% ±0.4%, *P*<0.01), whereas the apoptotic index was comparable between scramble shRNA-treated and control cells (Figure 4C). Parallel experiments using SK-Hep-1 cells revealed COMMD7 shRNA induced G2-phase arrest and raised apoptotic rate in those cells (Figure S1C and D).

### COMMD7 shRNA Retards HepG2 Cell Xenograft Growth In Vivo

**Figure 5:**
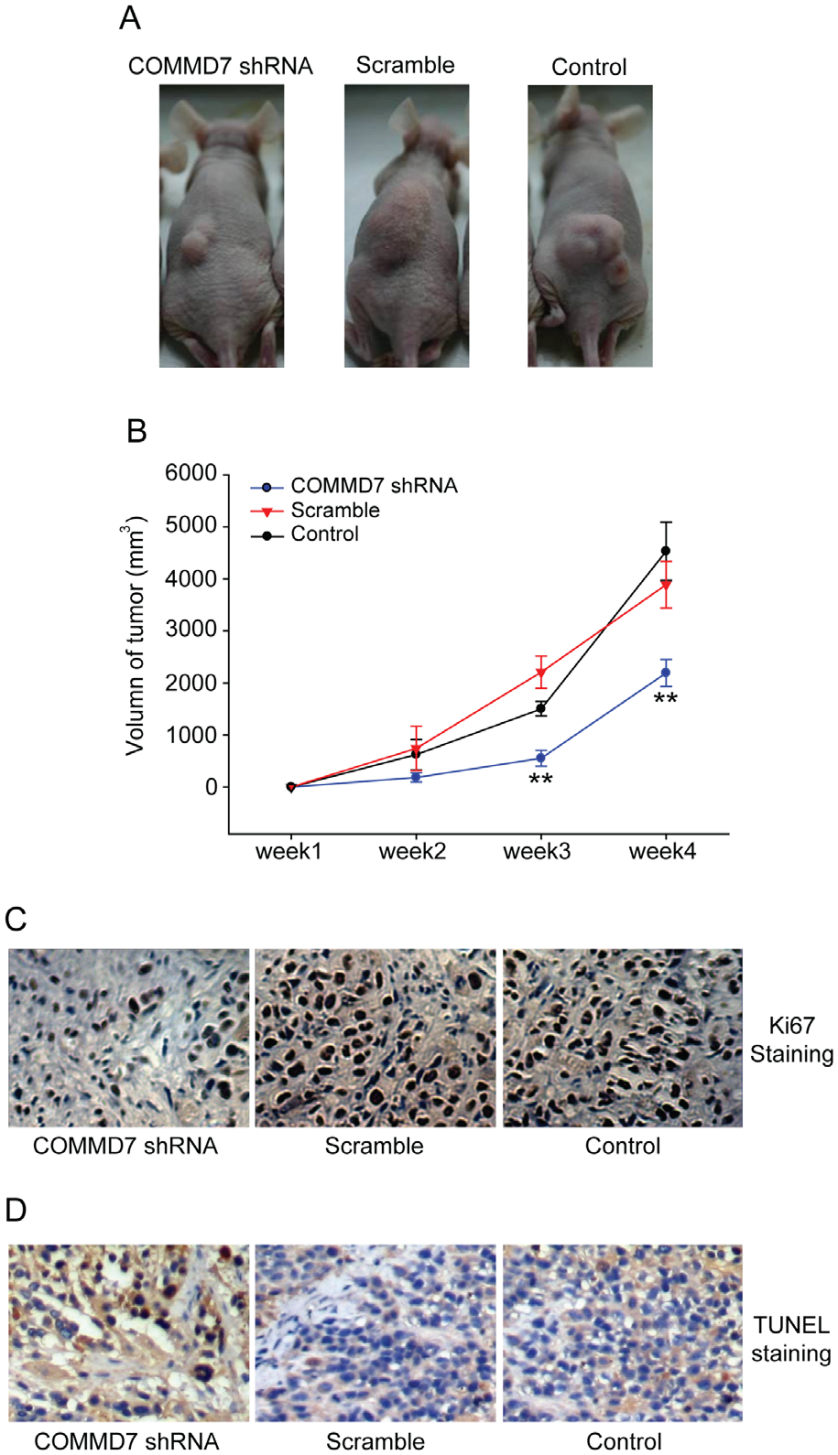
Effects of COMMD7 silencing on HepG2 cell tumorigenicity in nude mice. (A) Representative photographs of tumor xenografts. (B) Growth curves of tumor xenograft.^ **^ *P*<0.01 vs sramble shRNA treatment. (C) Ki67 staining was performed to show the markers for proliferation. (D)TUNEL staining showed the apoptotic cells.

Consistent with the in vitro experiments, COMMD7 shRNA significantly retarded the xenograft tumor growth in vivo as compared to control shRNA and blank control (Figure 5A). The measurements of xenograft tumor volumes showed that the size of tumor deriving from COMMD7 shRNA-treated cells was significantly smaller than those from control cells throughout four weeks of xenotransplantation (Figure 5B). Immunohistochemistry revealed less Ki67-positive tumor cells in the COMMD7 shRNA-treated group (Figure 5C), indicating COMMD7 shRNA inhibited cell proliferation in vivo. In addition, COMMD7 shRNA-expressed xenograft showed more cells underwent apoptosis (Figure 5D).

### COMMD7 shRNA Reduces NF-κB Signaling Responsiveness to TNF-α in HepG2 Cells

**Figure 6:**
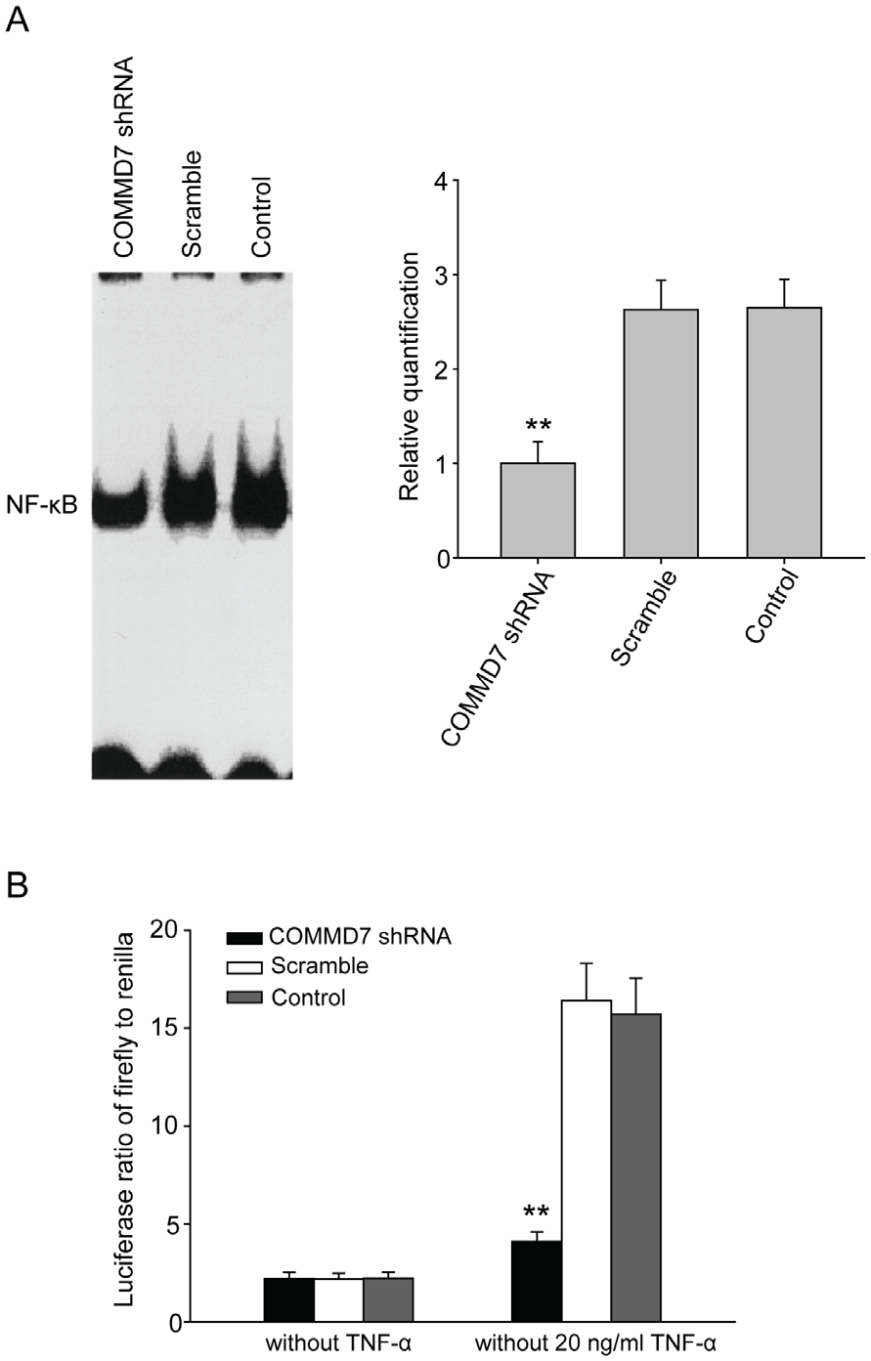
Effects of COMMD7 silencing on NF-κB signaling responsiveness to TNF-α. (A) EMSA analysis of NF-κB signaling. (B) Luciferase reporter assay. ^**^ *P*<0.01 vs sramble shRNA treatment.

EMSA revealed that the nuclear translocation of NF-κB in HepG2 cells was markedly impaired by COMMD7 shRNA although in the presence of TNF-α, compared to those in control cells (Figure 6A). The quantification of NF-κB signaling activity using luciferase reporter assay showed that the firefly/renilla luciferase ratio in COMMD7-silenced cells was only one quarter of those in control cells (Figure 6B).

## Discussion

Our preliminary studies show that COMMD7 is extensively overexpressed in multiple gastrointestinal malignancies, including HCC, gastric carcinoma, cholangiocarcinoma, and colon carcinoma. In this study, we further confirm that COMMD7 expression is aberrantly upregulated in HCC cell line, especially in HepG2 cells. We use loss-of-function experiments to further dissect the oncological role of COMMD7 in HCC. The knockdown using shRNA interference significantly down-regulates the endogenous expression of COMMD7 in HepG2 cells. The down-regulation of COMMD7 expression manifests as the reduction in cell proliferation, the increase in cell apoptosis, and the growth retardation of tumor xenograft in vivo. Moreover, equivalent inhibitory effects of COMMD7 shRNA on cell growth was also observed in another HCC cell lines, SK-Hep-1 cells, whose COMMD7 expression is comparable with HepG2 cells. In another aspect, COMMD7 overexpression in PLC/PRF/5 cells, whose COMMD7 expression is at the similar level as normal hepotocytes, increased cell proliferation (Figure S2A). These findings strongly suggest that COMMD7 contributes to the pathogenesis of HCC through its proliferative and/or antiapoptotic effects, especially in the sense of tumor progression as shown by our previous clinicopathological data [14].

The design and selection of siRNA sequence is critical for the efficiency of RNA interference (RNAi). In our preliminary experiment, three shRNA fragments were synthesized as suggested by Tuschl’s principle [20]. The analysis with Basic Local Alignment Search Tool identified no regions of local similarity between the designed sequence and any known sequence. The shRNA fragment that inhibits the transcription of COMMD7 gene at the rate of 80.2% is used for the subsequent COMMD7 knockdown, showing a high efficiency (49.4% on transient GFP fluorescence; 75% on real-time qRT-PCR).

Our MTT assay results confirm that COMMD7 shRNA significantly and specifically decreases the in vitro proliferation rate of viable HepG2 cells containing mitochondrial succinate dehydrogenase and capable of reducing extrinsic MTT. Additionally, the proliferation of cell population may be affected by cell-cell or cell-matrix interactions, in addition to the proliferative potential of individual cells in response to multiple environmental stimuli, including cytokines and growth factors. The colony formation assay, therefore, justifies the inhibition in the proliferative potential and environmental adaptation of individual COMMD7-knockdown HepG2 cells.

The suppression of HepG2 cell proliferation in vitro by COMMD7 shRNA can be the mixed contribution of arrested cell proliferation and activated apoptosis. Our flow cytometry results reveal that COMMD7 shRNA drives more HepG2 cells into the cycle arrest at S-phase, in contrast to less G2-phase cells ready for the subsequent mitosis. Interestingly, the effects of COMMD7 on cell cycle progression might be cell type-dependent, because COMMD7 shRNA-expressed SK-Hep-1 cells arrested at G2-phase. The exclusive roles of COMMD7 on cell growth in HepG2 and SK-Hep-1 cells are unlikely identical, and the potential mechanism, especially the control of cell cycle, need to be further studied. The additional apoptosis analysis with flow cytometry identifies an increased apoptosis in COMMD7-silenced HepG2 cells. It can be concluded that COMMD7 may be involved in the apoptosis resistance of HCC cells. Our in vivo tumor xenograft experiment also suggests that COMMD7 contributes significantly to tumor cell proliferation and apoptosis in vivo. However, COMMD7 overexpressed PLC/PRF/5 cells exhibited mild acceleration of cell cycle with no statistical significance (Figure S2B) and there is no significant difference of colony formation or cell apoptotic index between COMMD7-overexpressed PLC/PRF/5 cells and control cells, suggesting COMMD7 might play a minor role in the cellular activity of PLC/PRF/5 cells.

NF-κB has been known to be a multi-functional transcription factor [21]. Multiple cellular stimuli, such as TNF-α, enables the rapid translocation of NF-κB from cytoplasm to nucleus, activating the transcription of downstream genes [22]. It has been reported that NF-κB may be a potential mediator between chronic hepatitis and HCC, whose persistent activation is closely associated with the proliferation and metastasis of HCC cells [23]. The transfection of inhibitory IKKβ induced the specific suppression of IκB degradation and NF-κB activation in Mdr2-knockout murine hepatocytes [17]. The simultaneous depression of antiapoptotic factors, such as A1/Bfl1, c-IAP1 and GADD45β, initiated the apoptosis of HCC cells and eventually reduced the proliferation rate of HCC cells [17].

EMSA verifies the impairment in the nuclear translocation of NF-κB, and luciferase reporter assay further quantifies the inhibition rate (75%) of NF-κB in COMMD7-silenced HepG2 cells. These findings support that COMMD7 is likely to regulate the nuclear translocation of NF-κB and the consequent gene transcriptions involved in HCC growth. However, whether COMMD7 regulates the activation of NF-κB in a direct or indirect manner remains to be investigated.

### Conclusions

In conclusion, our study confirms the role of COMMD7, a newly identified HCC-related gene, in the growth of HCC cells. It is suggested that COMMD7 enhances HCC cell proliferation in vitro by overcoming cell cycle arrest and inducing apoptosis resistance. The proliferative and antiapoptotic effects of COMMD7 may result from the increased responsiveness of NF-κB signaling to TNF-α in the contest of HCC. COMMD7 is, therefore, a therapeutic target potentially useful for the improvement of HCC outcomes.

## Supporting Information

Figure S1 **Effects of COMMD7 silencing on SK-Hep-1 cells.** (A) MTT assay were performed at indicated days to show the proliferative curve. (B) Photographs and quantification of the colony formation assay. (C) Histogram of percentage of different cell cycle phase. (D) Apoptotic rates were analysed by Annexin V/FITC kit. ^*^ *P*<0.05; ^**^ *P*<0.01 vs sramble shRNA treatment.
(TIF)

Figure S2 **Effects of COMMD7 overexpression on PLC/PRF/5 cells.** (A) MTT assay were performed at indicated days to show the proliferative curve. (B) Apoptotic rates were analysed by Annexin V/FITC kit. ^*^ *P*<0.05 vs empty vector treatment.
(TIF)

Materials and Methods S1 (DOC)
