# Correction: ShRNA-Targeted COMMD7 Suppresses Hepatocellular Carcinoma Growth

**DOI:** 10.1371/journal.pone.0214259

**Published:** 2019-03-18

**Authors:** Lu Zheng, Ping Liang, Jing Li, Xiao-bing Huang, Shi-cheng Liu, Hong-zhi Zhao, Ke-qiang Han, Zheng Wang

Concerns were raised that some of the sequences reported in this article [1] may not correctly target the gene(s) of interest. To clarify this matter, the authors provide the following clarifications and corrections.

In Table 1, the sequences are written as 5’ to 3’, and for shRNA sequences, both rows in the first white band comprise a single sequence per column. The shRNA sequences were designed according to the polyclonal loci of pGenesil-1 vector, with the shRNA structure: BamHI+ Sense + Loop (TTCAAGACG) + Antisense+ Termination signal+ Sal I+ Hind III.

As noted in Table 1, the COMMD7 shRNA Sense sequence is as follows:

5’-GATCCG**CTCTGGGTCTTAGTGAGGA**TTCAAGACG**TCCTCACTAAGACCCAGAG**TTTTTTGTCGACA-3’

For clarity, the targeting (Sense, Antisense) sequences are in bold. The targeting sequence aligns to nucleotides 910–928 of COMMD7 transcript variant 1 mRNA sequence (NM_053041.2).

There is an error in Table 1 in that the COMMD7 shRNA Antisense sequence is reported in 3’ to 5’ orientation. The COMMD7 shRNA antisense sequence used in the study with targeting sequences in bold is as follows:

3’-GC**GAGACCCAGAATCACTCCT**AAGTTCTGC**AGGAGTGATTCTGGGTCTC**AAAAAACAGCTGTTCGA-5’

In Table 1, the Sense and Antisense sequences for COMMD7 (for PCR) are switched. The 5’ to 3’ sequence listed in Table 1 for COMMD7 Antisense (for PCR) is actually the Sense sequence, corresponding to nucleotides 758–779 of COMMD7 transcript sequence NM_053041.2. The sequence listed for COMMD7 Sense is actually the Antisense sequence, corresponding to nucleotides 938–915 of COMMD7 transcript sequence NM_053041.2.

A 2009 report [2] identified HepG2, used for many experiments in this article [1], as a cell line of hepatoblastoma rather than hepatocellular carcinoma origin. This may have implications for the conclusions regarding hepatocellular carcinoma biology: further experiments are needed to clarify whether results and conclusions of experiments using this cell line are generalizable to hepatocellular carcinoma.

In Fig 5, tumor sizes up to 5000 mm^3^ are reported. As noted in the Methods, tumor sizes were estimated using the equation, V = L x W^2^ (V = volume; L = length; W = width). This resulted in reported tumor volume estimates twice as large as would be calculated using the standard technique for this type of experiment in which tumor volumes are instead estimated as V = (L x W^2^)/2. [3, 4].

The authors also provide here additional information about the controls used for the reported experiments:

“Control” indicates HepG2 cells without plasmid transfection.

“Scramble” group was transfected with an shRNA construct generated using the following oligonucleotides:

(Sense) 5’-GATCC**GACTTCATAAGGCGCATGC**TTCAAGACG**GCATGCGCCTTATGAAGTC**TTTTTTGTCGACA-3’

(Antisense) 3’-G**CTGAAGTATTCCGCGTAC**GAAGTTCTGC**CGTACGCGGAATACTTCAG**AAAAAACAGCTGTTCGA-5’

The targeting sequence (bold) for the scramble construct includes a 14 nucleotide stretch that is 100% identical to the human ODF2 transcript (variant X34).

For electrophoretic mobility shift assays, the core κB binding sequence [5] in the reported NF-κB probe sequence is GGGACTTTCC.

The data underlying results in this article are no longer available.
