# Supplementary figures and images for: ShRNA-Targeted COMMD7 Suppresses Hepatocellular Carcinoma Growth
Source: PLoS One. 2012 Sep 25;7(9):e45412. doi: 10.1371/journal.pone.0045412 (PMC3458015; doi:10.1371/journal.pone.0045412)

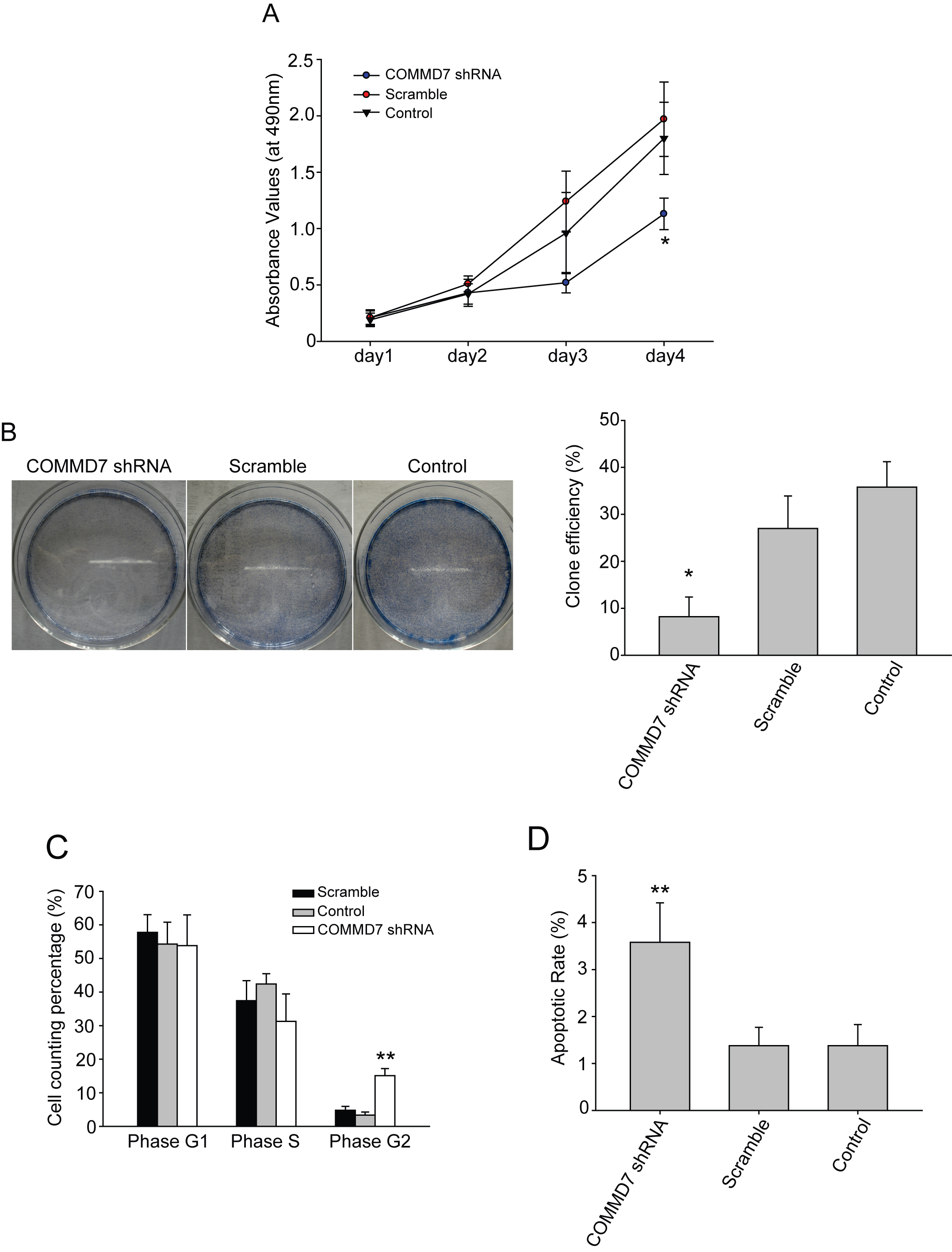

Supplement: Figure S1 — Effects of COMMD7 silencing on SK-Hep-1 cells. (A) MTT assay were performed at indicated days to show the proliferative curve. (B) Photographs and quantification of the colony formation assay. (C) Histogram of percentage of different cell cycle phase. (D) Apoptotic rates were analysed by Annexin V/FITC kit. * P<0.05; ** P<0.01 vs sramble shRNA treatment. (TIF) [file pone.0045412.s001.tif]

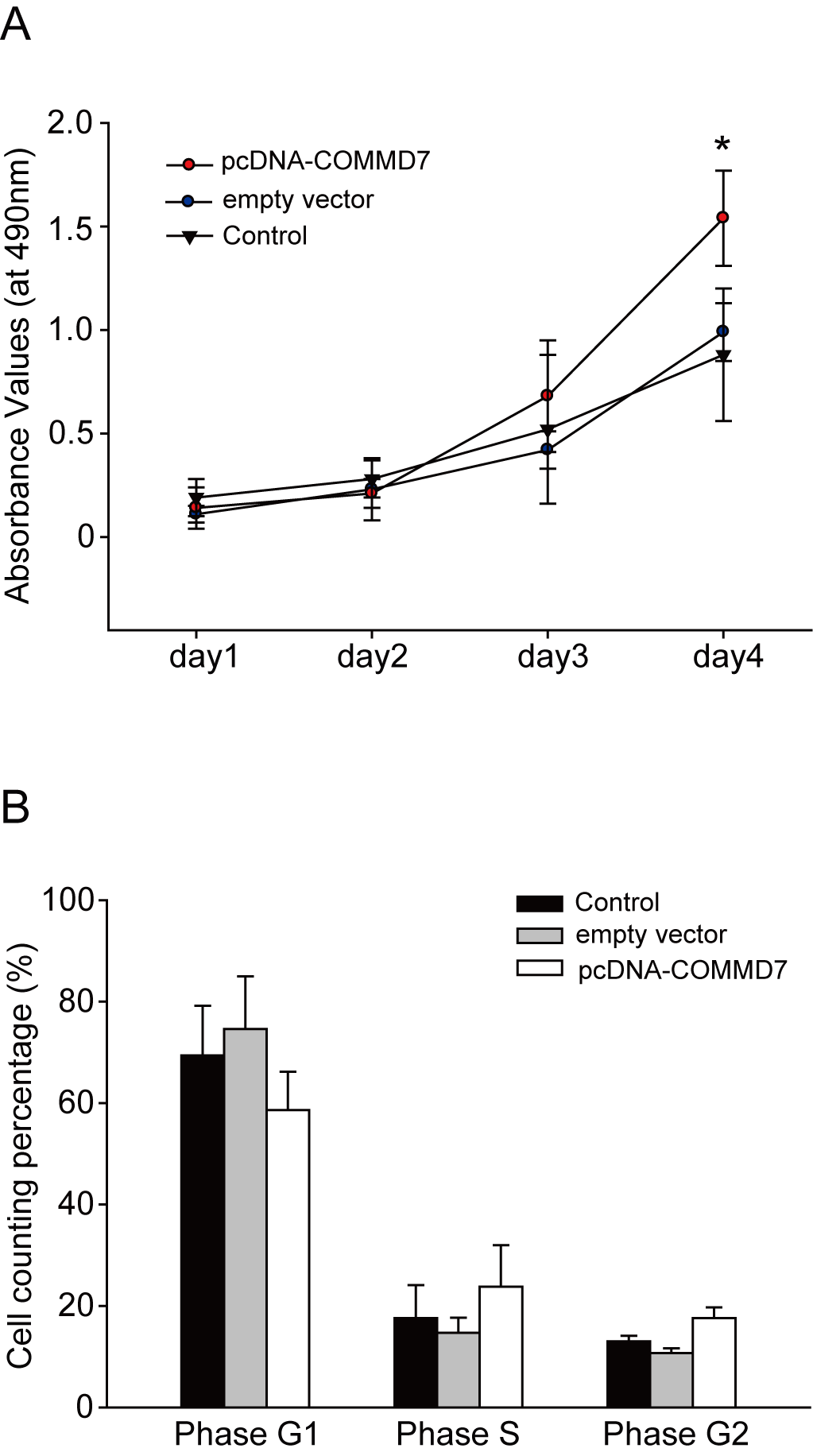

Supplement: Figure S2 — Effects of COMMD7 overexpression on PLC/PRF/5 cells. (A) MTT assay were performed at indicated days to show the proliferative curve. (B) Apoptotic rates were analysed by Annexin V/FITC kit. * P<0.05 vs empty vector treatment. (TIF) [file pone.0045412.s002.tif]
