# Supplementary material for: ShRNA-Targeted COMMD7 Suppresses Hepatocellular Carcinoma Growth
Source: PLoS One. 2012 Sep 25;7(9):e45412. doi: 10.1371/journal.pone.0045412 (PMC3458015; doi:10.1371/journal.pone.0045412)
Supplement: Materials and Methods S1 — (DOC) [file pone.0045412.s003.doc]

**Supporting Materials and Methods**

**Cell lines and cell culture**

HLE and HLF cells were obtained from Japanese Collection of Research Bioresources. SK-Hep-1 and PLC/PRF/5 cells were obtained from ATCC. HLF cells were cultured in RPMI 1640 containing 10% fetal bovine serum. Other three cell lines were cultured in DMEM containing 10% fetal bovine serum.

**COMMD7 silencing in SK-Hep-1 cells**

The generation of COMMD7 shRNA stably-expressed SK-Hep-1 cells was similar as that of HepG2 cells. Briefly, SK-Hep-1 cells were transfected with pGenesil-COMMD7-shRNA or scrambled shRNA by Lipofectamine™ 2000. Positive clones were selected in the presence of G418.

**Overexpression COMMD7 in PLC/PRF/5 cells**

COMMD7 cDNA was cloned into pcDNA3.1 (Invitrogen) named pcDNA-COMMD7. After sequencing analysis confirming the correction of clone, pcDNA-COMMD7 was transfected into PLC/PRF/5 cells and stably-expression cells were selected by G418.
